# Spinal Subarachnoid Hematoma After Spinal Anesthesia: A Case Report and Literature-Aligned Review

**DOI:** 10.7759/cureus.101930

**Published:** 2026-01-20

**Authors:** Mariana Machado, Patrícia Martins Lima, Cristiana Pinho, Margarida Barbosa

**Affiliations:** 1 Anesthesiology, Centro Hospitalar Universitário São João, Porto, PRT

**Keywords:** complication of neuroaxial anesthesia, neuroaxial anesthesia, neurological complication, spinal anesthesia, spinal subarachnoid hematoma

## Abstract

Spinal subarachnoid hematoma (SSH) is an infrequent but potentially devastating complication of spinal anesthesia (SA). Diagnosis may be challenging due to early nonspecific symptoms and the limited ability of magnetic resonance imaging (MRI) to reliably differentiate subarachnoid from subdural or epidural bleeding, making timely recognition critical. We report a 72‑year‑old woman who underwent uneventful SA for orthopedic surgery and initially recovered without complications. On postoperative day 2, she developed acute dorsolumbar pain progressing rapidly to complete bilateral lower‑limb paralysis and hypoesthesia below T12. MRI suggested a large posterior epidural hematoma extending from T9 to L2 with an inferior component interpreted as a subdural hematoma at L3-L4. Pharmacologic thromboprophylaxis had been initiated 24 hours after surgery in accordance with current European Society of Anaesthesiology and Intensive Care (ESAIC)/European Society of Regional Anaesthesia (ESRA) guidelines.

Urgent surgical decompression was performed approximately six hours after symptom onset. Intraoperatively, however, the hematoma was identified as an extensive SSH from T10 to L2, confirming a diagnosis that MRI had been unable to establish. A T10-L2 laminectomy with evacuation of the hematoma was completed successfully. Despite prompt recognition, guideline‑compliant anticoagulation timing, and rapid surgical intervention, the patient developed permanent neurological deficits.

This case illustrates how SSH likely requires the coexistence of substantial bleeding and anatomical conditions that restrict cerebrospinal fluid circulation, enabling clot formation within the subarachnoid space. Typical clinical presentation includes acute back pain followed by rapidly progressive motor, sensory, and sphincter dysfunction. Prognosis is strongly influenced by the severity of initial neurological impairment and the urgency of decompression, with optimal outcomes associated with surgery performed within 6-12 hours of deficit onset.

Given its rarity and potential for catastrophic outcomes even with protocol‑driven care, SSH must remain an important differential diagnosis in patients presenting with neurological deterioration after SA. This case reinforces the need for vigilant postoperative monitoring, immediate imaging when red‑flag symptoms arise, awareness of MRI limitations in compartmental differentiation, and timely multidisciplinary intervention.

## Introduction

Spinal subarachnoid hematoma (SSH) is an exceptionally rare but potentially devastating cause of acute spinal cord compression, particularly following atraumatic spinal anesthesia (SA) in patients without coagulation disorders. Reliable incidence data remain limited due to the scarcity of reported cases. Large national surveillance studies illustrate this rarity: for example, in Finland, only two cases of SSH were identified among approximately 1.4 million neuraxial blocks performed over a 10‑year period [1]. More broadly, spinal hematomas of any type are estimated to occur in roughly one in 220,000 neuraxial procedures, with subarachnoid hematomas representing only 15.7% of these cases [2]. Consequently, SSH remains an exceedingly uncommon entity, with most available knowledge derived from isolated case reports and small case series [2,3].

From a pathophysiological standpoint, SSH occurs when bleeding dissects into the subarachnoid space. Within this compartment, continuous cerebrospinal fluid (CSF) flow tends to dilute and disperse blood, which may explain the extremely low likelihood of significant hematoma formation [3,4]. Proposed mechanisms include injury to radiculomedullary vessels, inadvertent microtrauma during needle insertion, or bleeding from previously unrecognized vascular lesions, pathways that align with analyses of spontaneous SSH [5]. Although rare, SSH carries a high risk of long‑lasting neurological deficits, reinforcing the need for early recognition and timely intervention [2,3].

In this case report, we describe a 72‑year‑old woman who developed SSH following SA, highlighting the diagnostic challenges, interpretative limitations of magnetic resonance imaging (MRI), and the critical role of prompt surgical management.

This case was previously presented as a poster at the Euroanaesthesia Congress in 2021.

## Case presentation

The patient was a 72-year-old woman, American Society of Anesthesiologists (ASA) physical status classification II, scheduled for elective reduction and internal fixation of a right trimalleolar fracture. Past medical history included obesity, hypercholesterolemia, and hypertension. Medications were atorvastatin, lercanidipine, and a losartan-hydrochlorothiazide combination.

Preoperative evaluation confirmed a normal platelet count and coagulation profile, and the neurological examination revealed no abnormalities. SA was selected as the anesthetic technique for the procedure.

After standard ASA monitoring, the patient was positioned in right lateral decubitus. A spinal block was performed at the L3-L4 interspace using a 25‑gauge Quincke needle via a midline approach. After two attempts, free flow of clear CSF was obtained without blood. A mixture of hyperbaric 0.5% bupivacaine (10 mg) and sufentanil (2 µg) was injected into the subarachnoid space. No pain or neurological symptoms were reported during needle placement or injection.

Sensory block height was assessed using temperature testing and confirmed to reach the T10 dermatome bilaterally. The surgical procedure proceeded uneventfully. At the end of the surgery, the patient was transferred to the post-anesthetic care unit, where she remained until the neuraxial block was completely regressed.

On postoperative day 1, she had good pain control with systemic analgesia, was able to bear weight, and was ambulated with minimal assistance. Pharmacologic prophylaxis for deep‑vein thrombosis with enoxaparin 40 mg once daily was initiated 24 hours after surgery.

**Figure 1:**
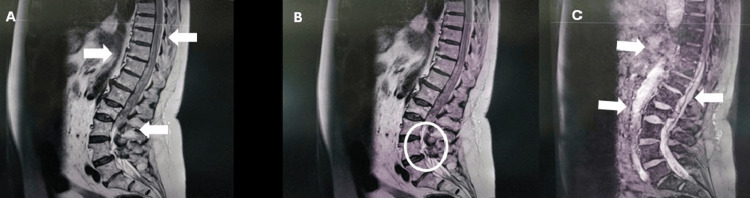
Sagittal MRI of the thoracolumbar spine demonstrating extensive spinal hematoma (A-B) Sagittal T1‑weighted images showing a large posterior epidural hematoma (white arrows) extending from T9 to L2, resulting in marked compression of the thecal sac with predominant involvement and anterior displacement of the conus medullaris. In B, the circled region highlights an inferior collection suggestive of a subdural component at L3-L4, associated with posterior deviation and crowding of the cauda equina nerve roots. (C) Sagittal T2‑weighted image confirming the craniocaudal extent of the epidural hematoma and the severity of resulting thecal sac compression (white arrows). Note the inferior high‑signal component corresponding to the suspected subdural extension. MRI: magnetic resonance imaging

On postoperative day 2, mild dorsolumbar discomfort rapidly progressed to severe, acute lumbar pain refractory to systemic analgesics and unrelieved by positional changes. Shortly thereafter, she became unable to ambulate. In accordance with the institutional protocol for complications following neuraxial anesthesia, the ward nurse immediately alerted the anesthesiology team. Neurological examination revealed complete motor paralysis of the lower extremities and hypoesthesia below the T12 dermatome, raising immediate concern for a compressive spinal lesion. The neurosurgical team was urgently consulted; intravenous dexamethasone 4 mg every eight hours was initiated, and an emergent MRI study was obtained. MRI showed a large posterior epidural hematoma extending from T9 to L2 with marked thecal sac compression and displacement of the conus medullaris. Inferiorly, at L3-L4, a distinct collection compatible with a subdural hematoma was identified with crowding and posterior deviation of the cauda equina (Figure 1). These findings correlated with the patient's rapidly progressive neurological decline and emphasized the importance of prompt recognition and accurate interpretation of spinal imaging.

**Figure 2:**
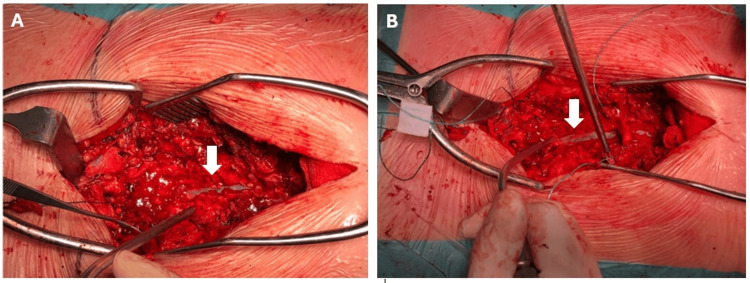
Intraoperative confirmation of an extensive spinal subarachnoid hematoma from T10 to L2 (A) Exposure of the spinal canal revealing the hematic collection prior to opening the arachnoid membrane; the arrow indicates the spinal cord displaced by the underlying hematoma. (B) After opening the arachnoid membrane, the arrow identifies the spinal cord directly compressed by the subarachnoid blood collection, providing the definitive diagnosis that could not be established by MRI alone. MRI: magnetic resonance imaging

Emergent surgical decompression was performed six hours after deficit onset under general anesthesia with standard ASA monitoring, invasive arterial pressure monitoring, and continuous depth‑of‑anesthesia assessment. Intraoperatively, an extensive SSH extending from T10 to L2 was directly visualized, establishing a diagnosis that could not be reliably determined through preoperative MRI alone (Figure 2). Although imaging suggested a large epidural hematoma with a possible inferior subdural component, MRI is inherently limited in differentiating subdural from subarachnoid blood collections. Following exposure of the spinal canal and opening of the arachnoid, the compressive subarachnoid collection was confirmed (Figure 2). A T10-L2 laminectomy with complete evacuation of the hematoma was performed. Postoperatively, the patient was admitted to the intensive care unit for close neurological and hemodynamic surveillance.

On the following day, the patient was transferred from the intensive care unit to the general ward, still exhibiting the preoperative neurological deficits, including persistent lower-extremity paralysis and hypoesthesia below the T12 level. At the three-month follow-up, she remained in a rehabilitation unit with ongoing lower-extremity paralysis, persistent paresthesia, urinary retention requiring catheterization, and fecal incontinence.

## Discussion

Spinal hematoma is a rare but severe complication of neuraxial anesthesia, including SA. Among locations, epidural hematoma is the most frequent and is typically attributed to needle‑induced trauma to the epidural venous plexus, whereas SSH remains exceptional; both its rarity and multifactorial causation are emphasized in meta‑analyses and national surveillance cohorts [2,6].

The principal etiological factors associated with SSH include coagulopathy/anticoagulant or antiplatelet therapy and iatrogenic causes, most commonly lumbar puncture for diagnostic or anesthetic purposes. In some patients, the coexistence of these factors further increases risk. SSH likely results from the interplay of coagulopathy, radiculomedullary vascular injury, and pre‑existing degenerative or stenotic anatomy that provides a locus minoris resistentiae within the canal [2,3]. Additional contributors include needle gauge/design, multiple attempts, and traumatic needle passage; technically difficult spinals are a recurrent pattern in the literature [7,8]. In rare instances, no predisposing factor is identified, and SSH occurs spontaneously [2,3].

In this case, two attempts with a 25-gauge Quincke needle may have incrementally increased the bleeding risk, despite atraumatic CSF return, an observation aligned with case-based reports [7,8].

Regarding bleeding risk, in this case, enoxaparin 40 mg once daily was commenced 24 hours after surgery, consistent with major guidelines for once‑daily pharmacologic thromboprophylaxis after a single‑shot spinal block, which advise administering the first postoperative dose more than 12 hours after neuraxial puncture (with longer intervals when clinically appropriate). The Joint European Society of Anaesthesiology and Intensive Care (ESAIC)/European Society of Regional Anaesthesia (ESRA) guidance further advises extending intervals after traumatic puncture and individualizing timing based on dose, renal function, and concomitant antithrombotics. Adherence reduces, but does not eliminate, neuraxial bleeding risk [9,10].

The most plausible mechanism underlying SSH is iatrogenic injury to radicular arteries or veins, resulting in blood entering the subarachnoid space. Continuous CSF circulation dilutes, disperses, and clears blood, limiting clot formation while permitting craniocaudal spread and nerve root clumping [3,4]. These CSF dynamics also help explain the rarity of SSH [3,4]. Blood may migrate across spinal levels and, rarely, extend intracranially, with secondary intracranial subarachnoid hemorrhage reported after SSH [11,12].

Consequently, SSH likely requires a combination of substantial bleeding and anatomical conditions that restrict CSF circulation, such as spondylosis, intervertebral disc herniation, or ligamentum flavum hypertrophy, to permit hematoma formation [3]. This pathophysiological substrate is also consistent with reported cases in which impaired CSF flow contributes to localized clot accumulation and longitudinal blood spread within the subarachnoid space [3,4].

This mechanism also explains the typical clinical presentation: acute back pain followed by rapidly progressive signs of spinal cord or cauda equina compression. Common manifestations include bilateral motor weakness or flaccid paralysis, hyporeflexia, hypoesthesia, and sphincter dysfunction, frequently urinary retention consistent with cauda equina syndrome [3,7]. In most published cases, SSH symptoms develop within two to four days after SA, reflecting the dynamic interaction between bleeding, CSF circulation, and neural compression [12].

The onset of neurological deficits following SA constitutes a medical emergency. Urgent MRI is the diagnostic standard for confirming hemorrhage, defining craniocaudal extent, and assessing the degree of cord/cauda compression. However, MRI may be unreliable in differentiating subdural from subarachnoid blood when collections are adherent to the arachnoid and meningeal planes are indistinct; in such situations, surgical exploration provides definitive compartmental diagnosis [3,13]. In our case, MRI suggested an epidural hematoma with an inferior subdural component, whereas intraoperative findings established SSH.

Conservative management of SSH has been successfully reported in selected cases presenting with minimal or rapidly improving deficits [11]. However, when severe or progressive signs of spinal cord or cauda equina compression are present, early diagnosis and prompt surgical decompression are essential to prevent permanent deficits [14].

Short‑course dexamethasone was used to mitigate pain and edema while arranging urgent decompression. Robust hematoma‑specific efficacy data are lacking; contemporary neuro‑emergency protocols prioritize the reversal of coagulopathy and rapid surgical decompression as definitive therapy. Steroids should therefore be considered a temporary adjunct rather than a disease‑modifying treatment [15,16].

Several factors have been associated with poorer outcomes following spinal hematoma, including delayed decompression, severe initial neurological deficits, rapid symptom progression, cervical involvement, extensive hematoma, and advanced age [3]. Among these, severe initial neurological impairment and sphincter dysfunction appear to be the strongest predictors of persistent long‑term deficits [3]. The beneficial impact of early decompression is well established, with significantly better neurological recovery when surgery is performed within 6-12 hours of deficit onset [14]. In this case, decompression occurred approximately six hours after the onset of neurological deterioration, aligning with these time‑critical recommendations.

The operative approach typically involves laminectomy, dural opening, and hematoma evacuation, with meticulous handling of neural structures to avoid additional iatrogenic injury [14].

This report underscores the importance of vigilant postoperative neurological monitoring following SA, immediate imaging when red flags arise, awareness of MRI limitations for compartmentalization, and guideline-concordant yet individualized antithrombotic timing, as well as time-critical decompression to mitigate the risk of permanent neurological deficits. Despite timely diagnosis and intervention in our patient, permanent deficits unfortunately still occurred, highlighting the potentially devastating severity of this condition.

This case also reinforces the value of institutional protocols for managing complications related to neuraxial anesthesia. Even when best practices and guideline-concordant, protocol-driven care are followed, such complications may still result in catastrophic outcomes. Therefore, early recognition and time-critical decompression remain essential to optimizing neurological recovery and minimizing the risk of permanent deficits.

Following this event, our institutional protocol was reviewed and optimized, and multidisciplinary team meetings were conducted to reinforce awareness of the protocol and emphasize adherence among all staff members.

## Conclusions

Although SSH following SA is exceedingly rare, it remains a significant and potentially serious complication, even when established safety guidelines are strictly followed. The emergence of red‑flag symptoms such as acute back pain, rapid neurological decline, or sphincter dysfunction should immediately raise concern for SSH, as delayed diagnosis is strongly associated with poorer neurological outcomes. Urgent MRI must be obtained without delay; however, clinicians should remain aware of its limitations in distinguishing subarachnoid from subdural or epidural bleeding. This reinforces the importance of maintaining a high index of suspicion, particularly when the degree of clinical deterioration appears disproportionate to radiological findings.

MRI remains central to diagnostic evaluation, but timely, protocol‑driven management, including prompt surgical decompression when indicated, continues to be the most critical factor in preventing irreversible neurological injury. Nevertheless, as demonstrated in this case, permanent deficits may still occur despite early recognition and intervention, underscoring both the severity of this condition and the essential role of rigorous institutional protocols that ensure early detection, rapid multidisciplinary activation, and vigilant postoperative neurological monitoring.

Given its rarity, this case provides meaningful clinical and epidemiological insight into neuraxial anesthesia-related complications and highlights the key learning points necessary to mitigate risk: close postoperative surveillance, heightened clinical awareness, careful consideration of procedural difficulty, and a low threshold for urgent imaging when neurological deterioration arises. Incorporating these strategies into routine practice may help reduce the morbidity associated with this uncommon but severe complication.
